# Risk Factors for Carbapenem-Resistant *Pseudomonas aeruginosa*, Zhejiang Province, China

**DOI:** 10.3201/eid2510.181699

**Published:** 2019-10

**Authors:** Yan-Yan Hu, Jun-Min Cao, Qing Yang, Shi Chen, Huo-Yang Lv, Hong-Wei Zhou, Zuowei Wu, Rong Zhang

**Affiliations:** Second Affiliated Hospital of Zhejiang University, School of Medicine, Hangzhou, China (Y.-Y. Hu, H.-W. Zhou, R. Zhang);; First Affiliated Hospital of Zhejiang University, School of Medicine, Hangzhou (Q. Yang);; Zhejiang Provincial Hospital of Traditional Chinese Medicine, Hangzhou (J.-M. Cao, H.-Y. Lv);; Hangzhou Third People’s Hospital, Hangzhou (S. Chen);; Iowa State University, Ames, Iowa, USA (Z. Wu)

**Keywords:** Carbapenem, risk factor, *Pseudomonas aeruginosa*, multidrug-resistant, surveillance, bacteria, China, antimicrobial resistance

## Abstract

Carbapenem-resistant *Pseudomonas aeruginosa* (CRPA) is a public health concern worldwide, but comprehensive analysis of risk factors for CRPA remains limited in China. We conducted a retrospective observational study of carbapenem resistance in 71,880 *P. aeruginosa* isolates collected in Zhejiang Province during 2015–2017. We analyzed risk factors for CRPA, including the type of clinical specimen; the year, season, and region in which it was collected; patient information, including age, whether they were an outpatient or inpatient, and whether inpatients were in the intensive care unit or general ward; and the level of hospital submitting isolates. We found CRPA was more prevalent among isolates from patients >60 years of age and in inpatients, especially in intensive care units. In addition, specimen types and seasons in which they were collected were associated with higher rates of CRPA*.* Our findings can help hospitals reduce the spread of *P. aeruginosa* and optimize antimicrobial drug use.

The bacterium *Pseudomonas aeruginosa* is a particularly concerning nosocomial pathogen because of its intrinsic resistance to multiple antimicrobial agents (*1*,*2*). In 2016, surveillance of nosocomial infections in China showed *P. aeruginosa* was the fifth most frequently isolated pathogen, accounting for 8.7% of hospital-acquired infections, and the fourth most common (8.0%) in Zhejiang Province (*3*,*4*). *P. aeruginosa* often causes severe infections and results in high rates of illness and death among infected patients (*1*). A survey in the United States revealed that *P. aeruginosa* was the second-leading cause of nosocomial pneumonia (14%–16%), third main contributor of urinary tract infections (7%–11%), and seventh major cause of bloodstream infections (2%–6%) (*5*,*6*). 

Carbapenems are the most effective antimicrobial agents against severe *P. aeruginosa* nosocomial infections involving bacteria producing cephalosporinase AmpC or extended-spectrum β-lactamases (*7*). However, *P. aeruginosa* has become increasingly resistant to carbapenems. A 2016 World Health Organization survey ranked carbapenem-resistant *P. aeruginosa* (CRPA) as the second most critical-priority bacterium among 20 antimicrobial-resistant bacterial species (*8*).

CHINET surveillance (http://www.chinets.com) revealed that CRPA in Zhejiang Province, China, increased annually from 22% in 2015 to 38.67% in 2017 and that Zhejiang had the highest rates of CRPA of all provinces in China in 2017. In addition, Zhejiang reported the local emergence of carbapenem-resistant *Klebsiella pneumoniae* carbapenemase–producing *P. aeruginosa* in 2015 (*9*). Given the clinical importance of CRPA, we analyzed short-term trends and various risk factors related to the occurrence of carbapenem resistance in *P. aeruginosa* in Zhejiang, as well as co-resistance to other commonly used antimicrobial agents. 

## Materials and Methods

### Bacterial Species and Strain Identification

We obtained data from the Annual Review of Hospital Infection Resistance Survey in Zhejiang Province, collected during 2015–2017 (*4*,*10*,*11*). Each of the >78 secondary or tertiary hospitals enrolled in the surveillance each year (Table 1) imported and shared data of routine antimicrobial susceptibility testing using WHONET 5.6 software (http://www.whonet.org). Enrolled hospitals are distributed in 11 cities of Zhejiang Province: Hangzhou, Huzhou, Jiaxing, Shaoxing, Ningbo, Taizhou, Jinhua, Quzhou, Lishui, Wenzhou, and Zhoushan. Each hospital laboratory cultured isolates on blood agar plates and identified antimicrobial-resistant strains by using matrix-assisted laser desorption/ionization time of flight (MALDI-TOF) mass spectrometry, the VITEK 2 Compact system (bioMérieux, https://www.biomerieux.com), or the Phoenix 100 system (Becton Dickinson, https://www.bd.com).

**Table 1:** *Pseudomonas aeruginosa* isolates obtained from hospitals in Zhejiang Province, China, 2015–2017

Year	No. hospitals*					No. isolates	Isolation rate, %†	Gram-negative isolates, %	Imipenem-resistant isolates, %
	Total	3A	3B	2A	2B				
2015	78	41	23	13	1	22,464	8.1	11.9	35.4
2016	88	44	23	19	2	24,303	8.0	12.0	37.1
2017	84	41	24	18	1	25,113	7.8	12.0	39.1
*Hospital classification is performed by the National Health Commission of China on the basis of the number of beds and comprehensive evaluation scores. Comprehensive evaluation covers the number of departments, staffing levels, management, technical level, work quality, and supporting facilities. Class 3 hospitals have >500 beds, class 2 hospitals have 100–499 beds. Grade levels are given on the basis of scores from a comprehensive evaluation; grade A hospitals received >900 points, grade B hospitals received 750–899 points. †*P. aeruginosa* was the fourth most commonly isolated pathogen in the region in each of the reported years.									

### Antimicrobial Susceptibility Testing

We performed antimicrobial susceptibility testing on 71,880 *P. aeruginosa* isolates submitted during 2015–2017. We tested for susceptibility to gentamicin, amikacin, piperacillin/tazobactam, ceftazidime, cefepime, aztreonam, imipenem, meropenem, ciprofloxacin, levofloxacin, colistin, and polymyxin B. We selected these 12 antimicrobial agents because all are used routinely in clinical settings in the province and we could include 1–2 from each antimicrobial category, per guidelines from the Clinical and Laboratory Standards Institute (CLSI; *12*). We imported susceptibility data into WHONET, deleted duplicated strains, used only the first isolate from each patient, and interpreted results according to CLSI guidelines (*12*).

Hospitals prepared isolates for susceptibility testing by using the Kirby-Bauer method and interpreted results manually according to CLSI guidelines (*12*) or by using broth microdilution for analysis by VITEK 2 or Phoenix 100 automated systems. To ensure comparable susceptibility tests between hospitals, each used the same reference strain, *P. aeruginosa* ATCC27853, and standardized procedures, following guidelines from the National Health Commission of China. We considered possible inaccuracies of susceptibility tests for colistin and polymyxin B in automated systems, especially by the Kirby-Bauer method, because of poor and slow diffusion in agar plates (*13*) and applied strict quality control practices by comparing results against our reference strain. 

We conducted imipenem susceptibility testing of 71,880 isolates and meropenem susceptibility testing of 26,916 (37.44%). We used imipenem resistance as an indicator of carbapenem resistance and separately analyzed imipenem-resistant (IMP-R) and imipenem-susceptible (IMP-S) *P. aeruginosa* isolates against the other antimicrobial agents.

### Classifications

We used year as an independent variant for occurrence analysis of IMP-R *P. aeruginosa.* Then, we calculated other variants by year. For our analysis, we categorized patient age into 6 groups: 0–2, 3–9, 10–19, 20–39, 40–59, and >60 years of age. Then we analyzed specific specimen types: blood, sputum, and urine. We analyzed outpatient and inpatient data and divided inpatients into 2 categories: those in intensive care units (ICUs) and those in standard patient wards (non-ICUs). To assess seasonality of CRPA, we analyzed quarters of the year, January–March, April–June, July–September, and October–December. 

We grouped hospitals into 4 levels, 3A, 3B, 2A, and 2B, according to classifications designated by the National Health Commission of China, which classifies hospitals on the basis of the number of beds and scores on a comprehensive evaluation. Class 3 hospitals have >500 beds, and class 2 hospitals have 100–499 beds. The National Health Commission grades hospitals using scores from a comprehensive evaluation of the number of departments, staffing levels, management, technical level, work quality, and supporting facilities. Grade A hospitals received >900 points; grade B hospitals received 750–899 points. 

**Figure 1:**
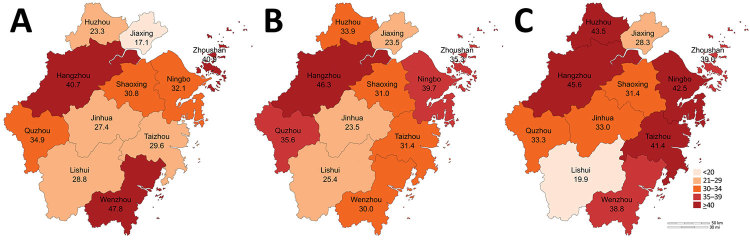
Heatmap of rates of carbapenem-resistant *Pseudomonas aeruginosa* each year in administrative districts in Zhejiang Province, China. A) 2015; B) 2016; C) 2017.

We grouped geographic regions by city (Figure 1). Then, we analyzed each variant by year (Appendix Figure 1). 

### Statistical Analysis

We analyzed antimicrobial resistance patterns of *P. aeruginosa* isolates exported from WHONET. We used unconditional logistic regression models to estimate odds ratios (ORs) and 95% CIs for univariable analysis of risk factors associated with IMP-R *P. aeruginosa*. We used either Pearson χ^2^ test or Fisher exact test to compare the frequency distribution of categorical variables. For all models, we considered p<0.05 statistically significant and then performed 2-sided probability on those results by using SPSS version 23.0 (IBM, https://www.ibm.com). We classified both intermediate and resistant isolates as IMP-R.

## Results

### Surveillance Data

Approximately 80 hospitals from 11 administrative districts in Zhejiang Province participated in the annual survey of antimicrobial resistance. *P. aeruginosa* was the fourth most frequently isolated nosocomial pathogen identified, accounting for 8.0% of all bacteria obtained and 12.0% of gram-negative bacteria collected in Zhejiang. During 2015–2017, hospitals submitted 71,880 *P. aeruginosa* isolates, >20,000 each year; this total is much higher than the numbers analyzed in studies from the United States and Europe (*14*,*15*). The large number of isolates provides a strong dataset for our statistical analysis. 

**Figure 2:**
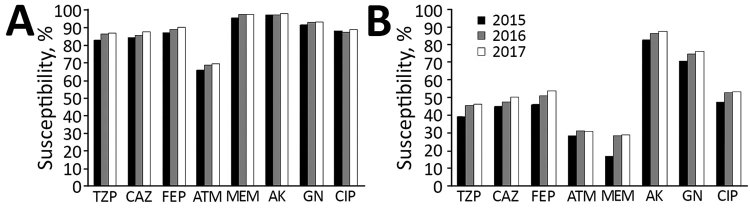
Annual susceptibility rates to antimicrobial agents among imipenem-susceptible (A) and imipenem-resistant (B) *Pseudomonas aeruginosa* isolates Zhejiang Province, China. AK, amikacin; ATM, aztreonam; CAZ, ceftazidime; CIP, ciprofloxacin; FEP, cefepime; GN, gentamicin; MEM, meropenem; TZP, piperacillin/tazobactam.

We found that 26,789 isolates (37.26%) were resistant to imipenem. The rate of IMP-R *P. aeruginosa* was >35% in each year and increased gradually during the study period. The meropenem resistance rate of ≈29% was slightly lower than that of imipenem resistance in the 3 years analyzed. In addition, we found that 29.54% of isolates were resistant to piperacillin/tazobactam and 25.11% were resistant to cefepime (Table 1; Figure 2; Appendix Table 1).

### Correlation of IMP-R *P. aeruginosa* with Risk Factors

We examined the correlation between IMP-R *P. aeruginosa* and risk factors by using OR (Table 2). We investigated quarter of the year, geographic region, patient age, inpatient or outpatient status, and ICU or non-ICU status as risk factors. Our analysis showed that the year isolates were collected had a statistically significant effect on the OR for IMP-R *P. aeruginosa*: OR 1.072 (95% CI 1.032–1.115) in 2016 compared with 2015 and OR 1.167 (95% CI 1.124–1.213) for 2017 compared with 2015. Seasonality was also a factor; *P. aeruginosa* isolates collected during January–March, April–June, and October–December were more likely to be IMP-R than those collected during July–September. We found that the capital of Zhejiang, Hangzhou, as well as Huzhou, Ningbo, Taizhou, Zhoushan, Wenzhou, and Quzhou, had a higher IMP-R *P. aeruginosa* rates than other cities. 

**Table 2:** Annual odds ratios for risk factors associated with carbapenem-resistant *Pseudomonas aeruginosa*, Zhejiang Province, China, 2015–2017*

Characteristics	2015			2016			2017	
	OR (95% CI)	p value		OR (95% CI)	p value		OR (95% CI)	p value
District								
Jiaxing	Referent			Referent			Referent	
Hangzhou	3.22 (2.85–3.63)	**<0.001**		2.83 (2.52–3.19)	**<0.001**		2.10 (1.91–2.31)	**<0.001**
Huzhou	1.42 (1.16–1.75)	**0.001**		1.68 (1.41–2.00)	**<0.001**		1.92 (1.55–2.38)	**<0.001**
Ningbo	2.23 (1.94–2.56)	**<0.001**		2.16 (1.89–2.47)	**<0.001**		1.85 (1.64–2.07)	**<0.001**
Taizhou	1.97 (1.65–2.36)	**<0.001**		1.50 (1.29–1.75)	**<0.001**		1.77 (1.51–2.07)	**<0.001**
Zhoushan	3.24 (2.63–4.00)	**<0.001**		1.79 (1.46–2.18)	**<0.001**		1.61 (1.31–1.97)	**<0.001**
Wenzhou	4.30 (3.75–4.94)	**<0.001**		1.40 (1.18–1.65)	**<0.001**		1.59 (1.40–1.80)	**<0.001**
Quzhou	2.99 (2.48–3.61)	**<0.001**		1.81 (1.53–2.14)	**<0.001**		1.25 (1.09–1.45)	**0.002**
Jinhua	1.77 (1.51–2.08)	**<0.001**		0.99 (0.85–1.15)	0.893		1.24 (1.09–1.40)	**0.001**
Shaoxing	2.09 (1.77–2.47)	**<0.001**		1..47 (1.26–1.71)	**<0.001**		1.10 (0.96–1.25)	0.165
Lishui	1.90 (1.59–2.27)	**<0.001**		1.11 (0.89–1.39)	0.345		0.62 (0.49–0.79)	**<0.001**
Specimen type								
Urine	Referent			Referent			Referent	
Blood	1.23 (0.99–1.53)	0.067		1.68 (1.35–2.08)	**<0.001**		1.44 (1.66–1.77)	**0.001**
Sputum	1.87 (1.66–2.96)	**<0.001**		1.97 (1.76–2.22)	**<0.001**		2.13 (1.90–2.39)	**<0.001**
Patient age, y								
0–2	Referent			Referent			Referent	
3–9	0.93 (0.58–1.49)	0.764		0.83 (0.56–1.23)	0.362		1.06 (0.73–1.54)	0.768
10–19	1.66 (0.99–2.48)	0.055		1.23 (0.84–1.80)	0.295		1.57 (1.08–2.29)	**0.018**
20–39	3.51 (2.48–4.97)	**<0.001**		2.28 (1.70–3.06)	**<0.001**		2.62 (1.95–3.55)	**<0.001**
40–59	3.93 (2.82–5.48)	**<0.001**		2.57 (1.95–3.39)	**<0.001**		3.09 (2.33–4.10)	**<0.001**
>60	4.34 (3.13–6.02)	**<0.001**		2.83 (2.15–3.71)	**<0.001**		3.24 (2.45–4.27)	**<0.001**
Quarter								
Jul–Sep	Referent			Referent			Referent	
Jan–Mar	2.11 (1.46–3.03)	**<0.001**		1.30 (1.17–1.44)	**<0.001**		1.90 (1.75–2.07)	**<0.001**
Apr–Jun	NA	NA		1.09 (0.97–1.22)	0.136		1.56 (1.42–1.70)	**<0.001**
Oct–Dec	NA	NA		1.28 (1.15–1.43)	**<0.001**		1.21 (1.11–1.31)	**<0.001**
Hospital level†								
2B	Referent			Referent			Referent	
2A	1.36 (0.71–2.63)	0.355		1.46 (0.96–2.20)	0.073		1.84 (1.11–3.04)	**0.016**
3B	1.13 (0.59–2.18)	0.712		0.95 (0.63–1.44)	0.819		1.35 (0.82–2.25)	0.239
3A	1.93 (1.01–3.71)	**0.044**		1.10 (0.72–1.68)	0.653		1.58 (0.95–2.63)	3.175
Type of patient								
Outpatient	Referent			Referent			Referent	
Inpatient, ward							1.15 (1.01–1.31)	**0.039**
Non-ICU	Referent			Referent			Referent	
ICU	2.60 (2.42–2.79)	**<0.001**		2.66 (2.49–2.85)	**<0.001**		2.57 (2.38–2.78)	**<0.001**

*Isolates from patients with missing values on the variables are not included in the analysis. Bold text indicates statistical significance. NA, not available; OR, odds ratio. †Hospital classification is performed by the National Health Commission of China on the basis of the number of beds and comprehensive evaluation scores. Comprehensive evaluation covers the number of departments, staffing levels, management, technical level, work quality, and supporting facilities. Class 3 hospitals have >500 beds, class 2 hospitals have 100–499 beds. Grade levels are given on the basis of scores from a comprehensive evaluation; grade A hospitals received >900 points, grade B hospitals received 750–899 points.

Isolates from inpatients had higher rates of imipenem resistance than those from outpatients, and isolates from patients in ICUs were more likely to be IMP-R than those from patients in non-ICU wards. When analyzed for patient age, the highest proportion of resistant isolates were collected from patients >60 years of age. We found no statistically significant difference in risk for IMP-R among isolates collected from patients 0–2 and 3–9 years of age. However, in other age groups, OR increased with age. In addition, we found that isolates from blood and sputum cultures were more likely to be IMP-R than isolates from urine (Table 2).

### Antimicrobial Resistance Patterns of *P. aeruginosa*

Overall, *P. aeruginosa* showed high susceptibility to lipopeptides (99.07% to colistin and 98.5% to polymyxin B) and aminoglycosides (93.06% to amikacin and 85.88% to gentamicin) but high resistance to cephalosporins and fluquinolones (≈20%–30% susceptibility) and aztreonam (35.65% susceptibility) (Table 3). When we classified isolates into IMP-R and IMP-S groups, we found statistically significant differences (p<0.001) in resistance rates between resistant and susceptible isolates for all analyzed antimicrobial drugs except lipopeptides. IMP-R isolates exhibited statistically lower susceptibility than IMP-S isolates to all antimicrobial drugs except the lipopeptides, colistin and polymyxin B. We saw a 2–3-fold difference in MIC_50_ (MIC needed to inhibit 50% of cells) between IMP-S isolates and IMP-R isolates. In contrast, for each antimicrobial drug except lipopeptides, most IMP-R strains belonged to the MIC_90_ group (MIC needed to inhibit 90% of cells), whereas the IMP-S isolates were more prevalent in the MIC_50_ group. Similarly, the IMP-R group was highly resistant (25.36%) to meropenem, but IMP-S group was highly susceptible (96.97%) (Figure 2).

**Table 3:** Antimicrobial resistance patterns of imipenem-resistant and imipenem-susceptible *Pseudomonas aeruginosa* isolates, Zhejiang Province, China, 2015–2017*

Antimicrobial drugs	No. isolates (susceptibility rate, %)		p value	Total susceptibility rate, %	MIC_50_, μg/mL			MIC_90_, μg/mL	
	IMP-S	IMP-R			S	R		S	R
Piperacillin/tazobactam	41,145 (85.70)	23,721 (44.01)	**<0.001**	70.46	8	64		64	128
Ceftazidime	30,326 (86.26)	18,348 (47.93)	**<0.001**	71.81	4	16		32	64
Cefepime	42,492 (89.01)	24,947 (50.83)	**<0.001**	74.89	2	8		16	64
Aztreonam	24,215 (68.07)	13,823 (30.32)	**<0.001**	54.35	8	32		32	64
Amikacin	42,106 (97.38)	24,748 (85.69)	**<0.001**	93.06	2	4		8	64
Gentamicin	41,207 (92.80)	24,618 (74.29)	**<0.001**	85.88	1	2		4	16
Ciprofloxacin	42,442 (88.28)	25,063 (51.64)	**<0.001**	74.67	0.25	1		2	4
Levofloxacin	41,982 (89.06)	24,593 (53.17)	**<0.001**	75.80	0.5	2		4	8
Meropenem	17,166 (96.97)	9,750 (25.36)	**<0.001**	71.03	1	8		1	16
Colistin	1,624 (99.08)	627 (99.04)	NA	99.07	1	1		1	2
Polymyxin B	5,012 (98.60)	3,746 (98.37)	0.452	98.50	1	1		2	2

*MIC_50_ and MIC_90_ were generated from the minimal inhibitory concentrations of antimicrobial drugs. Bold text indicates p values <0.05. IMP-R, imipenem-resistant; IMP-S, imipenem-susceptible; NA, not applicable; R, resistant; S, susceptible.

## Discussion

Carbapenems are the most effective antimicrobial agents against serious infections caused by multidrug-resistant gram-negative bacilli. However, the resistance rate of *P. aeruginosa* to carbapenems has been consistently high (*3*,*16*–*18*). Clarifying resistance trends of CRPA and related risk factors can guide antimicrobial use and selection of effective treatment plans.

In our study, rates of IMP-R *P. aeruginosa* increased annually and were higher in Zhejiang Province than reported for other provinces in national surveillance through CHINET (*3*,*17*,*18*). For instance, 2017 CHINET surveillance reported national rates of 27.3% for IMP-R *P. aeruginosa* and 25.1% for meropenem-resistant *P. aeruginosa* (*18*), but in Zhejiang Province the rates were 39.3% for IMP-R and 28.1% for meropenem-resistant isolates. Both the CHINET surveillance and our data indicated CRPA poses a severe challenge in Zhejiang Province. The slightly lower resistance rate we saw for meropenem could be because we tested fewer isolates for meropenem resistance (n = 26,916) than for imipenem resistance (n = 71,880) or could be the result of other mechanisms, such as mutation or loss of the oprD2 in some isolates (*19*).

When we examined risk factors, we found that patient type and ward were associated with a higher prevalence of IMP-R *P. aeruginosa*. Inpatients and those admitted to an ICU had higher IMP-R rates than outpatients and those in non-ICU wards, in accordance with previous studies (*20*), indicating ICU admission is a risk factor for IMP-R *P. aeruginosa.* Patient age also factors into IMP-R *P. aeruginosa* occurrence in Zhejiang (*21*), which could be a result of the low immune function of patients >60 years of age. We saw an increase in the rate of IMP-R with increased patient age but did not see increased rates for patients 0–2, 3–9, or 10–19 years of age. However, the IMP-R rate was >10% in 2015 and increased to 20.9% in 2017 in the 10–19-year age group (data not shown), which could signal a potential increasing trend of IMP-R in subsequent years. Further studies with clinical information and data are needed to investigate this issue.

A previous study in India showed that *P. aeruginosa* isolates from sputum and blood samples from patients in the ICU were more resistant than isolates from urine (*22*). Other studies in China also have observed this discrepancy of *P. aeruginosa* from various specimen types (*16*,*23*). We found this observation was true, not only for isolates from patients in the ICU but for all patient isolates included in our study, indicating IMP-R *P. aeruginosa* might be a less likely agent in urinary tract infection.

Previous studies also stated that the occurrence of *P. aeruginosa* infection was associated with seasons (*24*,*25*) and that the isolation rate usually was higher in summer than in winter. However, we observed a reverse outcome for IMP-R *P. aeruginosa*: a higher prevalence in winter than in summer (data not shown). The seasonal effect on IMP-R *P. aeruginosa* rates is unknown, but our finding could potentially inform clinical recommendations.

By OR analysis, we found that IMP-R *P. aeruginosa* was more prevalent in 7 administrative districts: Hangzhou, Huzhou, and Quzhou in the northwest and Ningbo, Taizhou, Zhoushan, and Wenzhou in the southeast of the province. However, we found no statistical differences in IMP-R related to hospital classification in Zhejiang, which is worth noting because patients in class 2 hospitals usually have mild or moderate illnesses and patients in class 3 hospitals have more severe conditions or are immunocompromised and more susceptible to infection. We weighted class 2 hospitals differently than class 3 hospitals in our statistical analysis to account for the difference in patient types. However, because we saw no statistically significant difference in imipenem resistance rates related to the hospital level, we should put the same weight on both classes of hospitals in future analyses.

Although our study showed *P. aeruginosa* was highly resistant to carbapenems and multiple other drugs, it remains highly susceptible to colistin and has some sensitivity to cephalosporins and fluoroquinolones. IMP-R *P. aeruginosa* is most sensitive to colistin in vitro, and colistin is effective against multidrug-resistant *P. aeruginosa* nosocomial infections (*26*). Despite its strong neurotoxicity and ototoxicity, colistin was reapproved for clinical applications in China in September 2017. However, efficacy of colistin monotherapy has been questioned in clinical trials (*27*), and colistin should be used in combination with other antimicrobial agents in clinical therapy. 

Novel antimicrobial agents approved by the US Food and Drug Administration, such as ceftolozane/tazobactam or ceftazidime/avivactam, could be other treatment options. These drug combinations have good efficacy against CRPA isolates (*28*,*29*) but currently are not approved for use in China. Of note, ceftolozane/tazobactam might not be useful against carbapenemase-producing *P. aeruginosa* (*30*), and prerequisite identification of resistance mechanisms would be needed to develop rational antimicrobial drug regimens. In addition, a novel plasmid-mediated colistin-resistant gene, *mcr*, has emerged in *Enterobacteriaceae* (*31*–*33*). To reduce the chances of its dissemination to *P. aeruginosa* under antimicrobial drug selection pressure, clinicians should prioritize colistin only for severe cases of *P. aeruginosa* infection in clinical practice. Because of limitations of susceptibility testing methods (*13*), MICs for polymyxins might be less reliable in strains with MICs close to the breakpoint. Therefore, clinicians also should choose polymyxin therapies carefully.

Our study had some limitations. We excluded strains without a corresponding field from the classification analysis, such as patient age, patient type, or isolation time, which might have caused a distortion in the resistance rate. A disproportionate number of class 3 to class 2 hospitals participated in the surveillance, and class 2 hospitals inevitably were biased in the statistical antimicrobial resistance rate because they submit fewer isolates. In addition, we could not include therapeutic regimens, patient outcomes, or the molecular mechanisms of resistance for CRPA strains because they were not available, but these measures could inform clinical decisions and should be included in further surveillance studies.

In summary, we conducted a comprehensive analysis of risk factors associated with CRPA in Zhejiang Province, China. We investigated potential risk factors for IMP-R *P. aeruginosa* because Zhejiang Province has higher rates of carbapenem resistance compared with other provinces (*34*). Our research provides insights into CRPA in China and indicates an imperative for medical institutions in China to strengthen surveillance for this organism.

Appendix Additional information on risk factors for *Pseudomonas aeruginosa*, Zhejiang Province, China, 2015–2017.
