# Supplementary material for: Risk Factors for Carbapenem-Resistant Pseudomonas aeruginosa, Zhejiang Province, China
Source: Emerg Infect Dis. 2019 Oct;25(10):1861–7. doi: 10.3201/eid2510.181699 (PMC6759267; doi:10.3201/eid2510.181699)
Supplement: Appendix — Additional information on risk factors for Pseudomonas aeruginosa, Zhejiang Province, China, 2015–2017. [file 18-1699-Techapp-s1.pdf]

Article DOI: https://doi.org/10.3201/eid2510.181699

Risk Factors for Carbapenem-Resistant *Pseudomonas aeruginosa*, Zhejiang Province, China

Appendix

Appendix Table. Surveillance for carbapenem-resistant *Pseudomonas aeruginosa* in hospitals, Zhejiang Province, China, 2015–2017*

Hospitals by city	Level†	Strain identification method‡	Years excluded§
Hangzhou			
First 17 People's Liberation Army Hospital	3A	VITEK 2 Compact	
Hangzhou Red Cross Hospital	3A	VITEK 2 Compact	
Hangzhou First People's Hospital	3A	MALDI-TOF MS	
Hangzhou Children's Hospital	3A	VITEK 2 Compact	
Hangzhou Hospital of Chinese Traditional Hospital	3A	Phoenix 100, VITEK 2 Compact	
Hangzhou Cancer Hospital	3A	VITEK 2 Compact	
Xixi Hospital of Hangzhou	3A	VITEK 2 Compact	
Sir Run Run Shaw Hospital, School of Medicine, Zhejiang University	3A	MALDI-TOF MS	
The Children's Hospital of Zhejiang University School of Medicine	3A	MALDI-TOF MS	
Women's Hospital, School of Medicine, Zhejiang University	3A	VITEK 2 Compact	
The First Affiliated Hospital of Medical School of Zhejiang University	3A	MALDI-TOF MS	
The Second Affiliated Hospital of Zhejiang University School of Medicine	3A	MALDI-TOF MS	
Hangzhou Second People's Hospital	3A	MALDI-TOF MS	
Zhejiang People's Armed Police Corps Hospital, Hangzhou	3A	Phoenix 100	
Xinhua Hospital of Zhejiang Province	3A	VITEK 2 Compact	
Zhejiang Provincial People's Hospital	3A	MALDI-TOF MS	
Zhejiang Provincial Hospital of Traditional Chinese Medicine	3A	MALDI-TOF MS	
Tongde Hospital of Zhejiang Province	3A	VITEK 2 Compact	
Zhejiang Hospital	3A	MALDI-TOF MS	
Zhejiang Cancer Hospital	3A	MALDI-TOF MS	
Hangzhou Third People's Hospital	3B	VITEK 2 Compact	
The First People's Hospital of Jiande	3B	VITEK 2 Compact	2017
First People's Hospital of Yuhang Distrcit, Hangzhou	3B	Phoenix 100	
Linan People's Hospital	2A	VITEK 2 Compact	2017
The First People's Hospital of Chunan	2A	VITEK 2 Compact	2015
Shulan Hospital	3A	Phoenix 100 system	2015, 2017
The First People's Hospital of Tonglu	2A	VITEK 2 Compact	2015
Huzhou			
The First People's Hospital of Huzhou	3B	VITEK 2 Compact	
Huzhou Central Hospital	3A	MALDI-TOF MS	2017
Changxing County People's Hospital	2A	VITEK 2 Compact	2017
Deqing People's hospital	2A	VITEK 2 Compact	2015
Jiaxing			
Jiaxing Maternity and Child Health Care Hospital	3A	Phoenix 100	
The Second Hospital of Jiaxing	3A	MALDI-TOF MS	
The First Hospital of Jiaxing	3A	MALDI-TOF MS	
Chinese medicine hospital of Jiaxing City	3A	VITEK 2 Compact	
The First People's Hospital of Jiashan	3B	VITEK 2 Compact	
The First People's Hospital of Pinghu	2A	VITEK 2 Compact	
The Second People's Hospital of Tongxiang	2A	VITEK 2 Compact	
Zhejiang Haining People's Hospital	3B	MALDI-TOF MS	2016
The First People's Hospital of Tongxiang	3B	VITEK 2 Compact	2016

Hospitals by city	Level†	Strain identification method‡	Years excluded§
Haiyan People's Hospital	2A	VITEK 2 Compact	
Haiyan County Chinese Medicine Hospital	2B	VITEK 2 Compact	2017
The Third Hospital of Jiaxing	2A	VITEK 2 Compact	2015, 2016
Shaoxing			
Shangyu People's Hospital	3B	VITEK 2 Compact	
The Second Hospital of Shaoxing	3B	VITEK 2 Compact	
Zhejiang Shengzhou People’s Hospital	3B	VITEK 2 Compact	
Zhuji People’s Hospital of Zhejiang Province	3B	VITEK 2 Compact	
Shaoxing Maternity and Child Health Care Hospital	3A	VITEK 2 Compact	
Shaoxing People’s Hospital	3A	VITEK 2 Compact	
Xinchang County People’s Hospital	3B	VITEK 2 Compact	2016, 2017
Ningbo			
The Affiliated Hospital of School of Medicine of Ningbo University	3A	MALDI-TOF MS	
Ningbo Women and Children’s Hospital	3A	VITEK 2 Compact	
Ningbo Medical Central Lihuili Hospital	3A	VITEK 2 Compact	
Ningbo Second Hospital	3A	VITEK 2 Compact	
Ningbo First Hospital	3A	MALDI-TOF MS	
Beilun People’s Hospital	3B	VITEK 2 Compact	
Maternal and Child Care Service Centre	3B	Phoenix 100	
People’s Hospital of Cixi City	3B	VITEK 2 Compact	
Yinzhou People’s Hospital	3B	MALDI-TOF MS	2015
Yuyao People’s Hospital of Zhejiang Province	3B	VITEK 2 Compact	2017
Fenghua People’s Hospital	2A	VITEK 2 Compact	
Ningbo No.7 Hospital	2A	VITEK 2 Compact	
Ningbo Fourth Hospital	3B	VITEK 2 Compact	2015, 2016
Taizhou			
Taizhou Municipal Hospital of Zhejiang Province	3B	VITEK 2 Compact	
The First People's Hospital of Taizhou	3B	VITEK 2 Compact	2017
Taizhou Hospital of Zhejiang Province	3A	MALDI-TOF MS	2015
Taizhou Central Hospital	3A	MALDI-TOF MS	2017
The First People’s Hospital of Wenling	3B	VITEK 2 Compact	
Yuhuan People’s Hospital	2A	VITEK 2 Compact	2015
Jinhua			
Dongyang People’s Hospital	3B	MALDI-TOF MS	
Jinhua Central Hospital	3B	MALDI-TOF MS	
The First People’s Hospital of Yongkang	3B	Phoenix 100	2015
Jinhua Municipal Central Hospital	3A	VITEK 2 Compact	
Lanxi People’s Hospital	2A	VITEK 2 Compact	
Panan People’s Hospital	2A	VITEK 2 Compact	
Pujiang People’s Hospital	2A	VITEK 2 Compact	
Wuyi First People’s Hospital	2A	VITEK 2 Compact	2015
Yiwu Central Hospital	3B	Phoenix 100	2015, 2016
Quzhou			
Quzhou People’s Hospital	3A	MALDI-TOF MS	
Jiangshan People’s Hospital	2A	Phoenix 100	
People’s Hospital of Changshan	2A	VITEK 2 Compact	2016, 2017
Longyou County People's Hospital	2A	VITEK 2 Compact	
Zhejiang Quhua Hospital	3B	Phoenix 100	2015
Quzhou Kecheng Hospital	2A	Phoenix 100	2015, 2016
Lishui			
Lishui People’s Hospital	3A	MALDI-TOF MS	2017
The Second People’s Hospital of Lishui	3A	VITEK 2 Compact	2015
Lishui Central Hospital	3A	VITEK 2 Compact	
Qingtian Country People’s Hospital	2A	VITEK 2 Compact	2017
Qingyuan County People's Hospital	2A	VITEK 2 Compact	2017
Jingning County People's Hospital	2B	MALDI-TOF MS	
Jinyun County People's Hospital	2A	VITEK 2 Compact	2015, 2016
Wenzhou			
The Second Affiliated Hospital of Wenzhou Medical College	3A	MALDI-TOF MS	
The First Affiliated Hospital of Wenzhou Medical College	3A	MALDI-TOF MS	
Wenzhou People's Hospital	3A	VITEK 2 Compact	
People’s Hospital of Yueqing City	3B	MALDI-TOF MS	
The People’s Hospital of Pingyang	3B	Phoenix 100	2015, 2017
The People’s Hospital of Cangnan	3B	VITEK 2 Compact	2015, 2016
Hospital of Integrated Traditional Chinese and Western Medicine of Wenzhou	3A	MALDI-TOF MS	2015, 2016
Zhoushan			
Zhoushan Hospital	3A	Phoenix 100	

Hospitals by city	Level†	Strain identification method‡	Years excluded§

*MALDI-TOF MS, matrix assisted laser desorption ionization-time of flight mass spectrometry.
†Hospital classification is performed by the Ministry of Health of China on the basis of the number of beds and comprehensive evaluation scores. Comprehensive evaluation covers the number of departments, staffing levels, management, technical level, work quality, and supporting facilities. Class 3 hospitals have >500 beds, class 2 hospitals have 100–499 beds. Grade levels are given on the basis of scores from a comprehensive evaluation; grade A hospitals received >900 points, grade B hospitals received 750–899 points.
‡ VITEK 2 Compact system (bioMérieux, https://www.biomerieux.com); Phoenix 100 system (Becton Dickinson, https://www.bd.com).
§Hospitals were excluded for years in which they submitted <3 isolates.

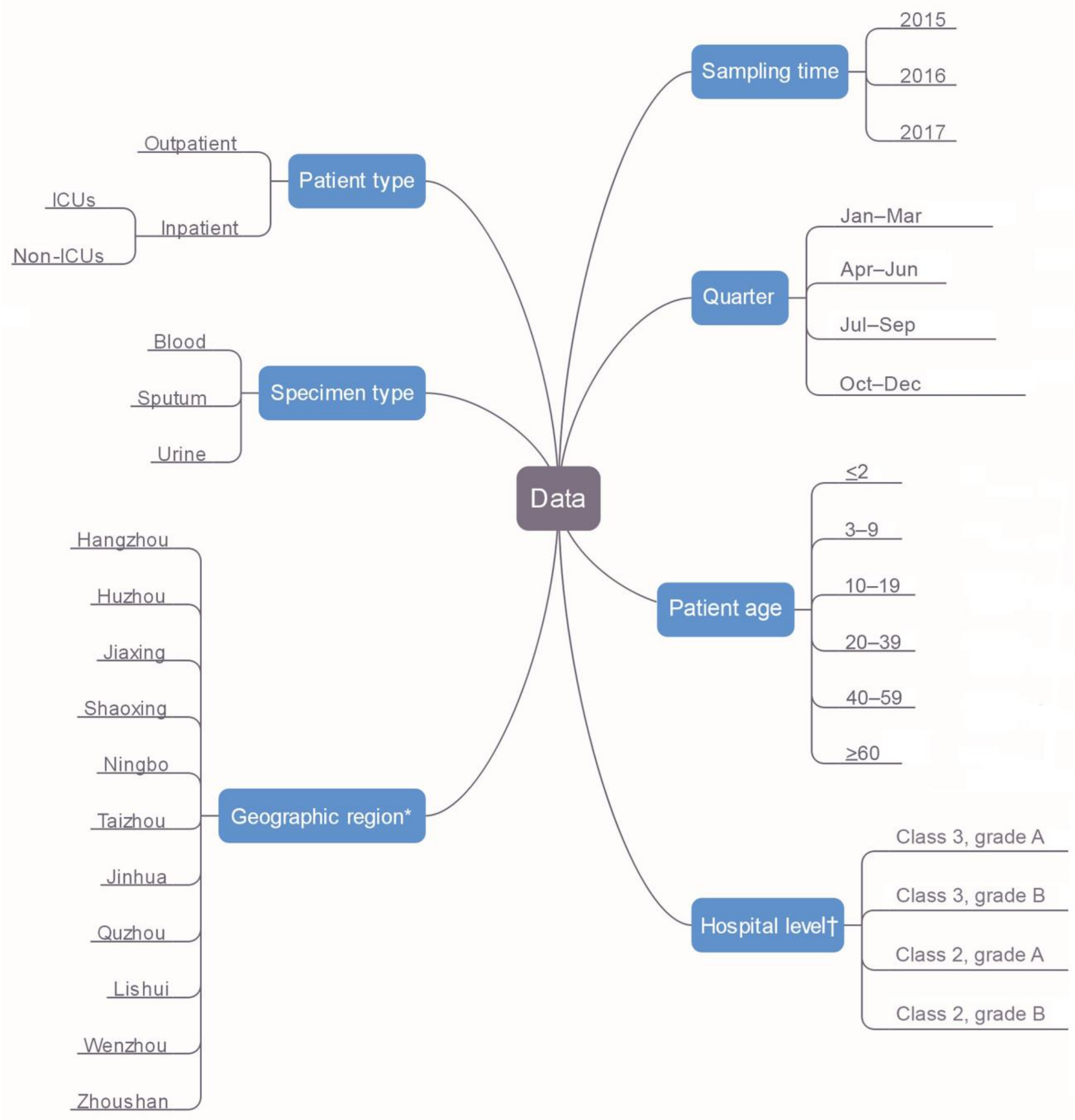

Appendix Figure. Diagram of data collected for determining risk factors for carbapenem-resistant *Pseudomonas aeruginosa* in Zhejiang Province, China, 2015–2017. ***** Administrative districts are designated by city names from the region. †Hospital classification is performed by the National Health Commission of China on the basis of the number of beds and comprehensive evaluation scores. Comprehensive evaluation covers the number of departments, staffing levels, management, technical level, work quality, and supporting facilities. Class 3 hospitals have >500 beds, class 2 hospitals have 100–499 beds. Grade levels are given on the basis of scores from a comprehensive evaluation; grade A hospitals received >900 points, grade B hospitals received 750–899 points.
